# Support-Free Low-Temperature Laser-Based Powder Bed Fusion of Polymers Using a Semi-Sintering Process

**DOI:** 10.3390/polym16233278

**Published:** 2024-11-25

**Authors:** Ryuichi Kobayashi, Takashi Kigure, Yuki Yamauchi

**Affiliations:** Tokyo Metropolitan Industrial Technology Research Institute, Tokyo 135-0064, Japan; kigure.takashi@iri-tokyo.jp (T.K.); yamauchi.yuki@iri-tokyo.jp (Y.Y.)

**Keywords:** low-temperature powder bed fusion, semi-sintering, laser sintering

## Abstract

In conventional laser-based powder bed fusion of polymers (PBF-LB/P), aging of the powder due to preheating of the powder bed is a significant issue. This paper proposes a method for low-temperature PBF-LB/P using a semi-sintering process that minimizes powder aging caused by preheating. By partially semi-sintering the low-temperature powder bed, it was possible to execute the PBF-LB/P while avoiding the aging of most of the powder. Furthermore, the suppression of curling by the semi-sintered body eliminated the need to connect the base plate to the parts, which was necessary in previously reported low-temperature PBF-LB/P. Using the semi-sintering process, we successfully built cuboid and tensile test specimens in a polyamide 11 powder bed maintained below the crystallization temperature, where the powder hardly aged. The apparent densities of the built specimens were comparable to those produced using high-temperature PBF-LB/P. However, the elongation in the building direction of the built parts by the semi-sintering process should be improved. This study represents the first step toward the practical application of low-temperature PBF-LB/P using semi-sintering.

## 1. Introduction

Laser-based powder bed fusion of polymers (PBF-LB/P) is one of the most suitable additive manufacturing techniques for the production of industrial components [1]. Powders that can be used in PBF-LB/P include polyamide (PA), polyethylene, polyether ether ketone, and polyphenylene sulfide [2,3,4]. Among these, polyamide powders PA12 and PA11 are the most commonly used [5]. In PBF-LB/P, the entire powder bed is typically preheated to a range known as the process window, which is often near its melting point [6]. Preheating prevents the melted regions formed by laser irradiation during the building process from fully solidifying and suppresses the curling of the melted regions. If the build is executed without preheating, curling will result in a loss of flatness of the powder bed. Consequently, during the recoating of the next layer of powder by the recoating mechanism, the powder bed will be disturbed, leading to a failure in the layering process. Therefore, preheating the powder bed is crucial. However, during the building process, the area of the preheated powder bed melted by laser irradiation is often less than 10%. This can be easily understood by considering common plastic products, in which the volume relative to their outer shape is not high. Thus, approximately 90% of the powder preheated in the machine is recovered without melting. The recovered powder (aged powder), which is preheated to near its melting point, exhibits characteristics different from those of the virgin powder [7,8,9]. Unfortunately, these changes are often disadvantageous for PBF-LB/P.

It has been reported that building with a powder prone to aging can deteriorate the surface quality (known as orange peel) and mechanical properties of the produced component [10,11,12]. Such quality degradation occurs in inkjet-printing-based PBF methods such as multi-jet fusion [13,14]. Polymer powders for PBF are expensive, and as previously mentioned, a large amount of aged powder is generated after a single build. Therefore, the aged powder is typically not discarded. Instead, it is often blended with the virgin powder, with the blend ratio commonly requiring at least 30% virgin powder [15,16,17,18,19]. This blend ratio (at least 30%) exceeds the powder consumption per build (approximately: 10–20%); therefore, the generation of excess powder is unavoidable.

The fundamental cause of excess powder generation is powder aging due to the preheating of the powder bed. To avoid powder aging, several low-temperature PBF-LB/P methods have been proposed, in which the preheating temperature of the powder bed is set lower than usual. In low-temperature PBF-LB/P, the powder bed temperature is set below the crystallization temperature of the polymer powder. Niino et al. successfully implemented a rigid base plate on a PBF-LB/P apparatus and connected the base plate to parts with support structures to suppress curling during low-temperature building using PA12 [20]. In another study, Niino et al. succeeded in achieving low-temperature PBF-LB/P for polyetheretherketone, a high-performance engineering plastic [21]. Recently, Antończak et al. proposed the construction of polylactide using low-temperature PBF-LB/P with a dual laser beam. Similar to the study conducted by Niino et al., the first layer of the parts was fixed to the base plate [22].

In terms of further advancement, Schlicht et al. proposed support-free low-temperature PBF-LB/P (i.e., without connecting parts to the base plate with support structures) [23,24,25]. With their method, they successfully built 10 mm cubes, small springs, and tensile test specimens using fractal quasi-simultaneous exposure strategies. To the best of our knowledge, currently, the largest built object using support-free low-temperature PBF-LB/P is the tensile test specimen. To build the tensile test specimen, its longitudinal direction was positioned at an angle greater than 30° relative to the horizontal direction of the powder bed. When the angle was set to more than 30°, the slice data area calculated from the 3D model of the tensile test specimen was relatively small, resulting in the maximum width of the laser scan area per layer being less than 20 mm. This small-slice data area was selected to avoid the effects of curling and warping, thereby facilitating the building process. Moreover, their study found that the elastic modulus of the polypropylene tensile specimens fabricated using low-temperature PBF-LB/P was lower than those of specimens fabricated using high-temperature PBF-LB/P. Numerous voids were observed on the fracture surface of the polypropylene specimen, leading to the qualitative conclusion that the presence of localized voids influenced the elastic modulus.

**Figure 1:**
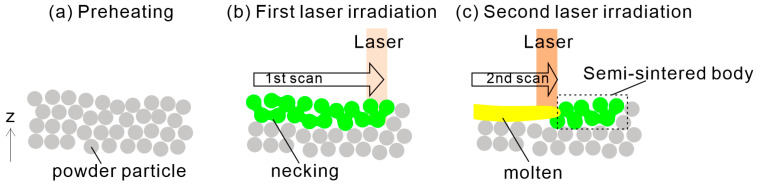
Schematic of low-temperature PBF-LB/P using a semi-sintering process. In this process, (**a**) Preheating, (**b**) First laser irradiation, and (**c**) Second laser irradiation are sequentially performed within the same layer.

Support-free low-temperature PBF-LB/P is still in its infancy and requires further exploration. Schlicht et al. suppressed curling in support-free low-temperature PBF-LB/P by homogenizing the temperature of the irradiation area through fractal scanning of the laser. Different from this, we propose low-temperature PBF-LB/P using a semi-sintering process that physically suppresses curling. Previously, the semi-sintering process was proposed to suppress the formation of a rough surface called “orange peel” [26]. Figure 1 shows a schematic of the semi-sintering process modified for low-temperature PBF-LB/P. As shown in Figure 1a, the powder bed is preheated below the process window (i.e., below the crystallization temperature of the polymer powder). The proposed method involves two laser irradiations with different energy densities on the same layer. The first irradiation involves laser exposure to partially sinter the powder bed (Figure 1b), and the second irradiation melts a portion of the partially sintered powder (Figure 1c). This process eliminates the need to preheat the entire powder bed to near its melting point, thus avoiding much of the powder from preheating-induced aging. In addition, the semi-sintered body, in which the powder particles are necked together, exhibits some strength which enables it to physically suppress the curling of the molten portion. Furthermore, by incorporating anchors to build parts and reverse the correction of warping, support-free low-temperature PBF-LB/P can be realized.

## 2. Materials and Methods

The PBF-LB/P apparatus (RaFaEl300F, Aspect, Tokyo, Japan) used in our experiments was equipped with a fiber laser with a wavelength of 1.06 µm. This apparatus was used in its standard PBF-LB/P configuration with no special hardware modifications made for the purposes of this study. The powder used was PA11 powder (PA11, ASPEX-FPA (black), Aspect, Tokyo, Japan). In the experiments, the thickness of the powder layer was consistently set to 0.1 mm. The building chamber was purged with nitrogen.

### 2.1. Aging Evaluation of PA11 Powder

To determine the temperature range for low-temperature PBF-LB/P with PA11, builds without laser irradiation (i.e., part cake manufacturing without parts) were prepared at various powder bed temperatures. In this experiment, virgin PA11 powder was used, and a 30 mm high build without laser irradiation was used. Since the powder bed for PA11 was preheated to a range of 180–187 °C in previous studies [27,28,29,30], the powder bed temperature was set to a wider range in our experiments, with the specific temperatures being 23 (no heating by heater), 100, 150, 170, 180, 185, and 190 °C. After building was completed without laser irradiation, the powder was collected from the part cake. The bulk densities and melting points of the collected powders were measured. First, the powder was gently poured into a stainless steel container with a capacity of 100 cm^3^, and the weight of the powder was measured using an electronic balance. The bulk density was calculated by dividing the weight by the volume of the container. The experiment was performed in triplicate, and the average value was calculated. The heat flow was measured using differential scanning calorimetry (DSC) with a sample weight of 7.0 mg, at a heating rate of 10°/min. After the measurement, the peak melting point was obtained using the evaluation software provided with the apparatus. Measurements were conducted on five samples, and the average values were calculated.

### 2.2. Exploring Laser Parameters for Semi-Sintering Process

Based on the results of the powder aging evaluation (Section 3.1), a powder bed temperature of 150 °C was selected for the experiment with the semi-sintering process. To determine the appropriate laser parameters for the semi-sintering process, laser irradiation with different energy densities was performed on a 10 mm × 10 mm area of the powder bed preheated to 150 °C. Table 1 presents the conditions used to determine suitable laser parameters for the semi-sintering process. The laser energy densities in Table 1 were calculated by dividing the laser power by the scan speed and pitch. After laser irradiation, the building chamber was cooled to below 100 °C before being exposed to the atmosphere. The powder bed was then imaged.

### 2.3. Building Cuboid Specimens

**Figure 2:**
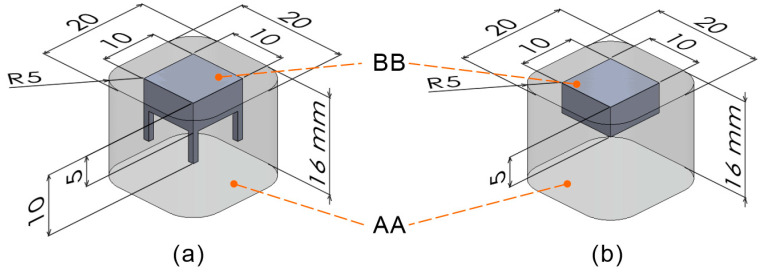
Three-dimensional model for building the cuboid specimen: (**a**) cuboid with anchors, (**b**) cuboid without anchors.

To evaluate the quality of the parts built using the proposed process, cuboid specimens with dimensions of 10 mm × 10 mm × 5 mm were fabricated. Figure 2 shows the 3D model loaded into the PBF-LB/P apparatus for the specimen construction. Figure 2a,b show the models with and without anchors, respectively. The AA model, with outer dimensions of 20 mm × 20 mm × 16 mm (for semi-sintering body fabrication), overlapped with the BB model, which included a 10 mm × 10 mm × 5 mm cuboid and anchors (for specimen fabrication). Regarding the placement of components during building, the AA model was built at least 10 mm from the base plate of the building chamber. The laser irradiation parameters for the AA model were selected from the s-4 conditions in Table 1 based on the results described in Section 3.2. Table 2 presents the laser irradiation parameters for the BB model. Three specimens were constructed for each condition, as listed in Table 2. After building, the semi-sintered body (excluding the area from AA to BB) was removed using a scraper, followed by a blasting treatment. The side surfaces of the specimens were imaged after blasting. Anchors were added to further improve curl suppression and were ultimately unnecessary. After removing the anchors with nippers, the apparent density of the cuboids was measured using the Archimedes method. Moreover, to confirm the semi-sintered state between the powder particles, a semi-sintered body built under the s-4 conditions was observed by scanning electron microscopy (SEM). For the observation, the semi-sintered body was gold coated. For the powder that was not irradiated by the laser during building, the powder bulk density and melting point measurements were made as described in Section 2.1.

### 2.4. Building Tensile Test Specimens

**Figure 3:**
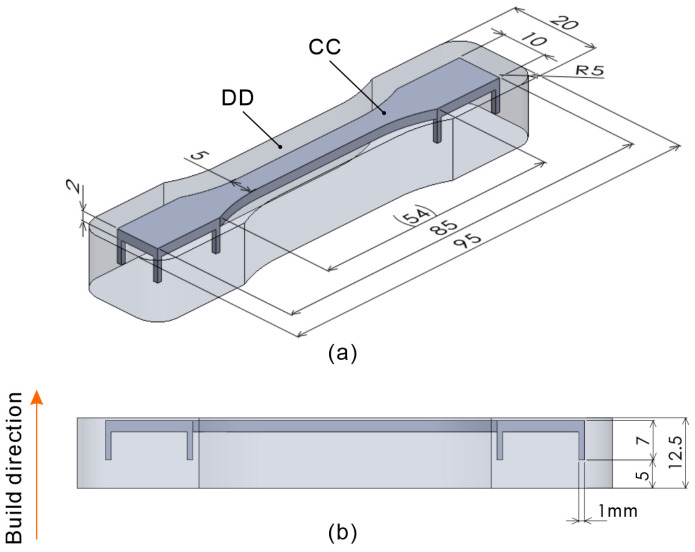
Three-dimensional model showing the basic shapes of the tensile test specimen and semi-sintered body: (**a**) isometric view, (**b**) front view.

**Figure 4:**
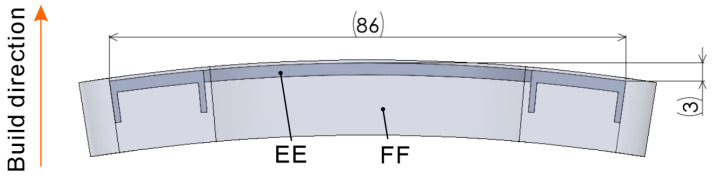
Three-dimensional model with warpage correction.

Figure 3 shows the basic shapes of the tensile test specimens used in this study. Figure 3a shows the isometric view, and Figure 3b shows the front view. The CC model is a body for melting, and the DD model is a body for semi-sintering. Preliminary experiments using the proposed process, which aligned the 2 mm dimension in Figure 3a with the build direction, revealed significant warping of the tensile test specimens during the build. Although there were instances where the build was successfully completed by chance, interference with the recoating mechanism and specimen frequently occurred. Therefore, a warpage correction was performed on the basic shape, as shown in Figure 4. The correction amount was determined by measuring the warpage from the few specimens that were successfully produced using the model shown in Figure 3. The shape was then remodeled using the bend function in the 3D CAD software (SOLIDWORKS, 2023 SP5.0) to reverse the warpage.

**Figure 5:**
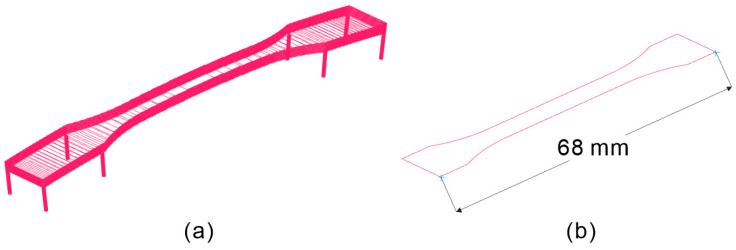
Slices calculated from body EE: (**a**) stack, (**b**) widest slice.

Figure 5a shows slices calculated at intervals of 0.1 mm from body EE. Among the slices shown in Figure 5a, the widest slice is shown in Figure 5b. Its width was 68 mm, which was thrice the length reported in previous studies on support-free, low-temperature PBF-LB/P [25].

**Figure 6:**
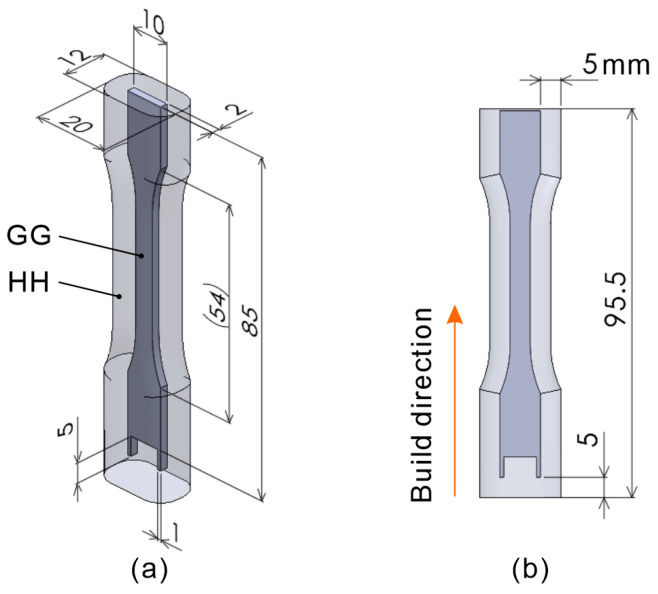
Three-dimensional model of a tensile test specimen with its build direction and longitudinal direction aligned: (**a**) isometric view, (**b**) front view.

Moreover, tensile test specimens built in the longitudinal direction aligned with the build direction, as shown in Figure 6. The tensile test specimen bodies CC, EE, and GG were built under three conditions, namely, m-5, m-7, and m-9, as listed in Table 2, whereas the semi-sintered bodies DD, FF, and HH were built under conditions s-4, as listed in Table 1. Furthermore, in one building, one of the 3D models EE and FF was built at the same horizontal position in the building chamber, but at different vertical positions. In other words, some of the tensile specimens were fabricated by stack building.

### 2.5. Evaluation of Tensile Test Specimens

For the tensile test specimens built using the 3D model with the warpage correction shown in Figure 4, 3D scanning was performed after building to evaluate the effect of the correction. A sublimation-type antireflective spray was applied to the tensile test specimens for 3D scanning. The 3D scanner (FRALE Pro 16M, TOKYO BOEKI Techno-System, Tokyo, Japan) had an average resolution of 0.033 mm per pixel. The shape of the warp was visualized by obtaining the cross-sectional profile of the test specimen at the position passing through the center of the 10 mm dimension in Figure 3 from the scanned data.

Tensile tests were performed using a universal testing machine (Autograph AG-10 kN, Shimadzu, Kyoto, Japan). For each building condition (m-5, m-7, and m-9), three specimens were tested. The anchors of the tensile specimens were cut before mounting them on the universal testing machine. The crosshead speed was set to 2.0 mm/min.

X-ray CT scans were performed to visualize the voids within the tensile test specimens. However, to eliminate the influence of the tensile test on the voids, samples were obtained from other tensile test specimens (i.e., these samples were not tensile tested) which were constructed under the same conditions. The voxel sizes were 5 µm × 5 µm × 12 µm. The resulting tomographic image was a composite of 200 tomographic slices (i.e., a thickness of 1 mm). A composite image was generated by selecting the voxel with the lowest intensity from 200 voxels at the same position in the thickness direction. This method allowed for the representation of all voids within a 1 mm depth in a single image, thereby emphasizing the voids. Furthermore, the composite images were evaluated for voids using image processing software (ImageJ, 1.54g). The procedure involved extracting an area of approximately 3.85 mm × 2.82 mm in the center of the specimen from the composite images of each condition. After noise removal using median filtering, these images were binarized with the same threshold, and the number of voids, their average size, and their maximum size were calculated.

To further analyze the tensile test results, DSC curves were obtained for the remnants of the tensile-tested specimens. For the interpretation of the result, the DSC curves of the part cake built at 150 °C and the semi-sintered bodies built under the s-4 condition (Table 1) were obtained. The heating rate was 10 °C/min, and the sample weighed 7.0 mg.

## 3. Results and Discussion

### 3.1. Powder Aging Caused by Differences in Powder Bed Temperature

**Figure 7:**
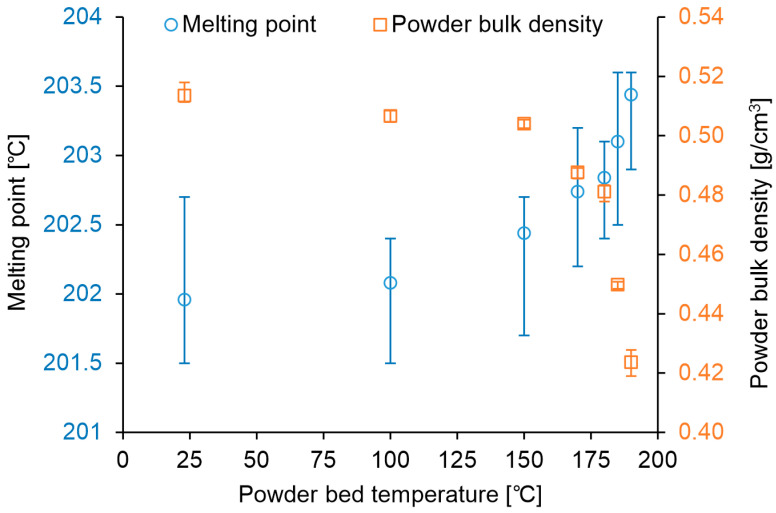
Relationship between the powder preheating temperature and changes in powder properties.

Figure 7 shows the relationship between the powder bed temperature, powder melting point, and powder bulk density. The error bars indicate the maximum and minimum values. From 23 °C to 150 °C, the powder bulk density gradually decreased, and the melting point increased. However, beyond 150 °C, these changes became more pronounced.

**Figure 8:**
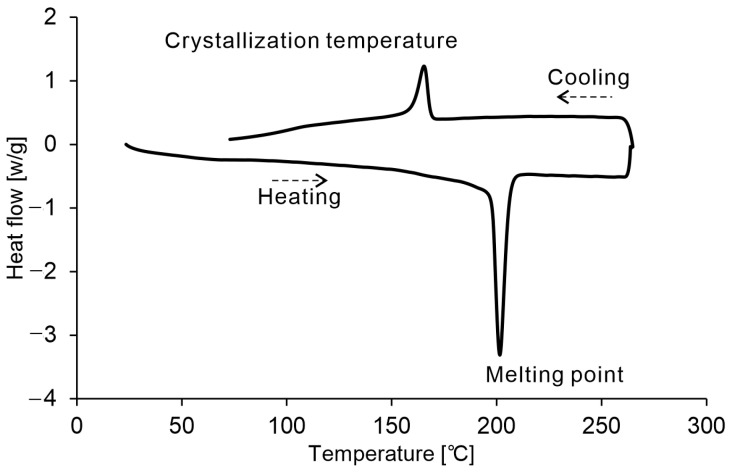
DSC curve of virgin PA11 powder.

Figure 8 shows the DSC curve of the virgin PA11 powder. While 150 °C is on the lower side for the crystallization temperature, 170 °C falls between the crystallization temperature and the melting point (i.e., within the process window). Therefore, preheating within the process window can induce changes in the powder characteristics. Based on these results, the upper-limit temperature for the low-temperature PBF-LB/P of PA11 used in this study was determined to be 150 °C.

### 3.2. Laser Irradiation Suitable for Semi-Sintered Body Manufacturing

**Figure 9:**
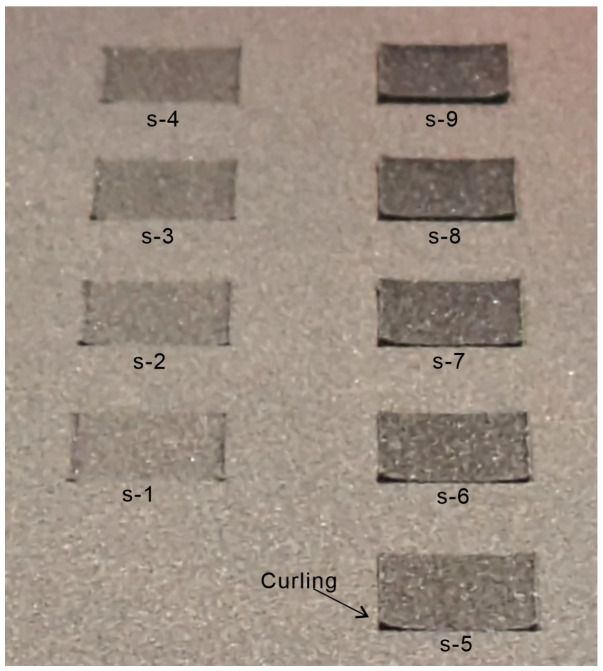
Image of the powder bed after laser irradiation.

Figure 9 shows an image of the powder bed after laser irradiation at various energy densities on a 150 °C powder bed. The reason for not selecting a lower temperature was that the greater the temperature difference between the powder bed and the melting point, the more significant the effects of curling and warping. The areas irradiated by the laser exhibited a color change, making them easily visible. The conditions under which curling did not occur were those with energy densities lower than condition s-4. Under conditions s-5 and beyond, it is believed that much of the powder melted and shrank, leading to curling. Therefore, for semi-sintered bodies, conditions under or below s-4 were considered appropriate. Furthermore, in this study, it was desirable for a semi-sintered body to have sufficient strength to suppress curling and warping. Hence, s-4, which exhibited the highest laser energy density within an appropriate range, was selected.

### 3.3. Evaluation of Cuboid Building

**Figure 10:**
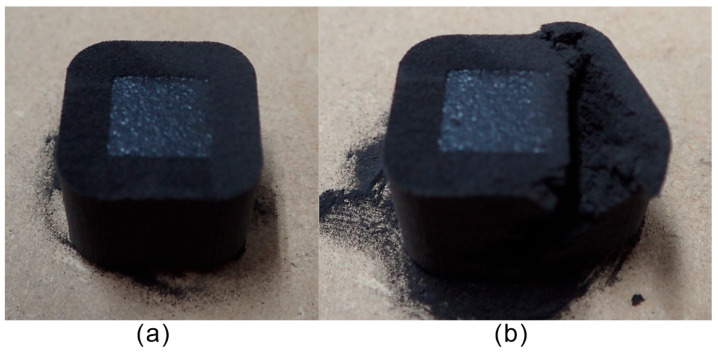
Cuboid specimen with a semi-sintered body: (**a**) as built, (**b**) during the removal of semi-sintered body.

Figure 10 shows the images of a cuboid specimen with a semi-sintered body constructed using the proposed method. Figure 10a shows the specimen harvested from the part cake. Figure 10b shows the removal of the semi-sintered body. Although the semi-sintered body had sufficient strength to avoid being considered a powder, it was still manageable enough to be removed manually.

**Figure 11:**
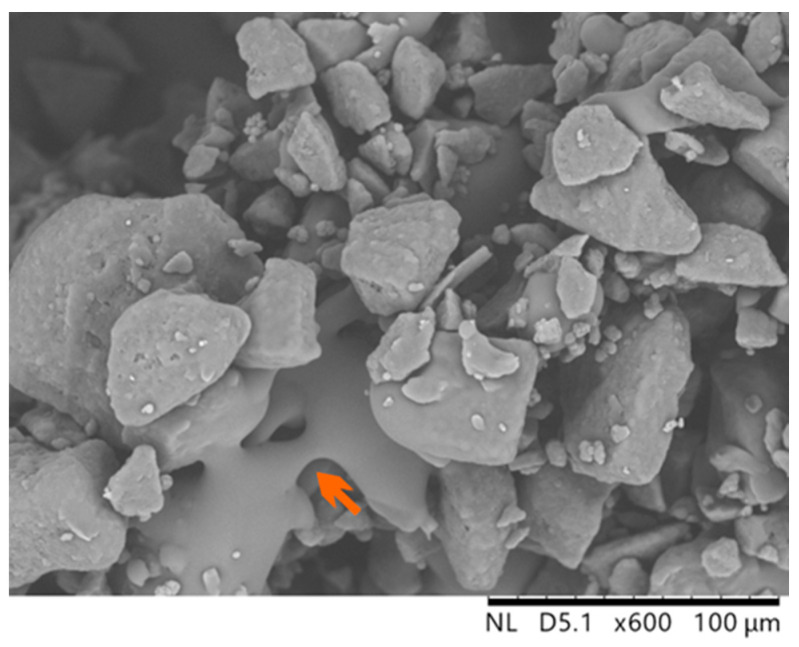
SEM image of the semi-sintered body; the arrow indicates the necking formation between the powder particles.

Figure 11 shows an SEM image of the semi-sintered body. As indicated by the arrows in the figure, necking of the adjacent powder particles due to slight melting was observed. This necking is believed to have contributed to the slight increase in the strength to suppress curling.

**Figure 12:**
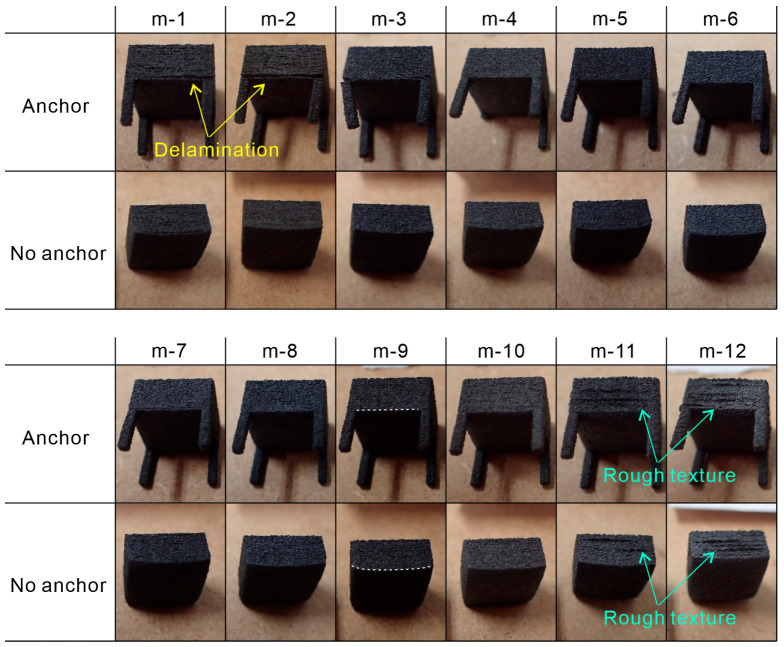
Images of cuboid specimens built under various laser parameters.

Figure 12 shows images that qualitatively demonstrate the effects on the sides of the cuboid specimen and the impacts of anchors. In the images under laser irradiation conditions m-1, m-2, and m-3 with anchors, delamination was observed at the boundary between the anchor and the cuboid, whereas no delamination was observed without anchors. In the m-9 image, white dotted lines were drawn to enhance the visibility of the bottom shape of the cuboid, clearly indicating that the bottom without anchors was more warped. Therefore, the parts without anchors exhibited deformation due to curling or warping; however, the addition of anchors resisted these deformations. Under conditions of low laser energy density, delamination occurred because the built part could not withstand the forces attempting to deform it. However, from condition m-4 onward, no clear delamination was observed, suggesting that a sufficient energy supply can prevent delamination. This indicates that although the proposed process is feasible without anchors, the presence of anchors improves the flatness of the bottom surface. Under conditions m-11 and m-12, rough textures were formed on the sides of the cuboid specimen, likely due to melting caused by excessive heat input through thermal conduction. The laser irradiation conditions that produce this type of rough texture are incompatible.

**Figure 13:**
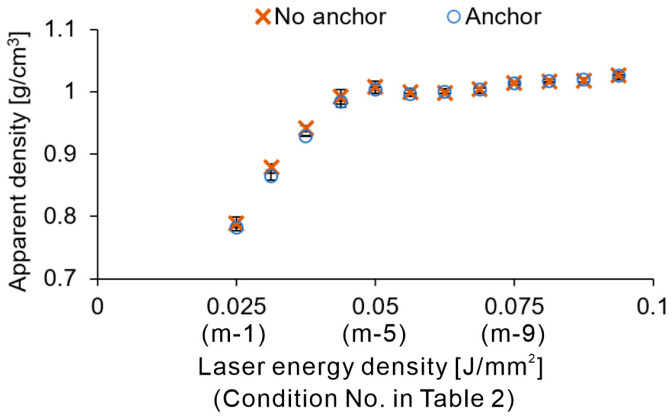
Apparent density of cuboid specimens with and without anchors.

Figure 13 shows the density measurements of the cuboid specimens. The apparent density reached approximately 1.00 when the laser irradiation energy density was 0.05 (under the m-5 condition). The apparent density of the parts built using the virgin PA11 powder was approximately 1.05 g/cm^3^ [10], which was close to that observed in high-temperature PBF-LB/P. In addition, the density values under the m-6 and m-7 conditions were slightly lower than those under m-5. Beyond m-7, a slight increase in the density was observed. This upward and downward behavior of the apparent density with increasing laser energy density may be attributed to the combination of polymer thermal decomposition and melting due to thermal conduction. The behavior of the apparent density in previous studies on laser absorption in powder beds was consistent with the density change trends observed in this study [31]. Based on these results, conditions with a laser energy density lower than m-4 were deemed unsuitable. By combining the results of the side texture evaluation and the apparent density evaluation of the cuboid specimens, we selected the following build conditions for the tensile test specimens: m-5, m-7, and m-9.

The bulk density of the unmelted powder harvested from the part cake after building the cuboid specimens was 0.508 g/cm^3^ (mean of *n* = 3), and the melting point of the powder was 202.6 °C (mean of *n* = 5). In the build with a powder bed at 150 °C without laser irradiation, depicted in Figure 7, the bulk density of the powder was 0.504 g/cm^3^, and the melting point was 202.4 °C. For comparison, in the build with a powder bed at 185 °C, depicted in Figure 7, the bulk density of the powder was 0.450 g/cm^3^, and the melting point was 203.1 °C. The evaluation values of the unmelted powder harvested from the part cake after building the cuboid specimens were close to the results of the 150 °C powder bed without laser irradiation, indicating that the impact of powder aging due to laser irradiation was minimal.

### 3.4. Evaluation of Tensile Test

**Figure 14:**
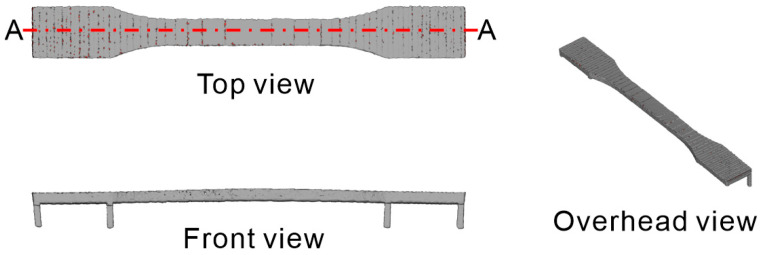
Representative images of the 3D scan data of the tensile specimen. A–A shows the location where the effectiveness of warp correction was evaluated.

**Figure 15:**
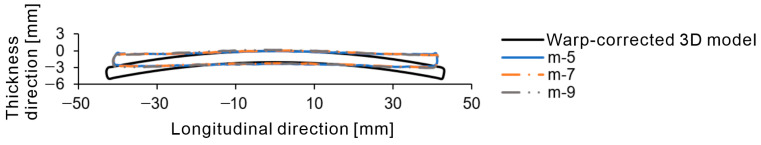
Cross-sectional view of the 3D scan data of tensile test specimens.

Figure 14 shows a representative example of a 3D scan of a tensile test specimen built using the slice data of the warp-corrected 3D model EE. Figure 15 includes the graph plots of the cross section at positions A–A in Figure 14, along with a comparative cross-sectional plot at the same position derived from the warp-corrected 3D model EE. The effectiveness of warp correction was confirmed under all conditions when compared with the corrected model. Furthermore, the cross-sectional data for m-5, m-7, and m-9 were largely identical, indicating that the amount of warp correction was independent of the laser conditions.

**Figure 16:**
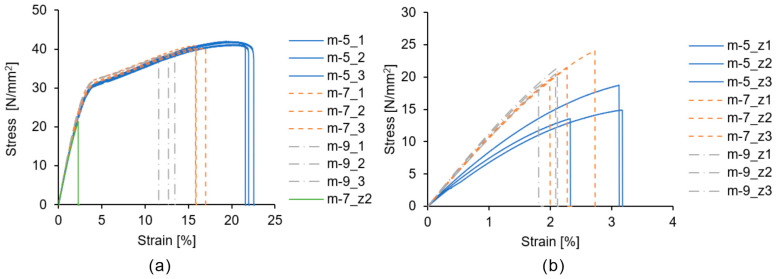
Tensile test results: (**a**) stress–strain curves of specimens built using 3D model EE (note that m-7_z2 shows the results of the 3D model GG for comparison), (**b**) stress–strain curves of specimens built using the 3D model GG.

Figure 16 shows the stress–strain curves obtained from the tensile tests. Figure 16a shows the test results for the tensile specimens built using the 3D model EE. Figure 16b shows the test results for the tensile specimens built using the 3D model GG. Note that in the figure legend, _1, _2, _3 correspond to the three test results of the EE specimens, and _z1, _z2, _z3 correspond to the three test results of the GG specimens. For ease of comparison, one of the test results for m-7 in Figure 16b is included in Figure 16a. Under all the build conditions, the tensile specimens built using the 3D model EE exhibited a consistent elastic behavior with strains below 3% and a yield point of approximately 4%. The average elongation at break was 22.0% for m-5, 16.2% for m-7, and 12.5% for m-9, indicating that the specimens elongated more with the decrease in the laser irradiation energy density. Tensile specimens were built using the 3D GG model, in which the longitudinal and build directions were aligned, and these specimens fractured before reaching the yield point. The average elongations at break were 2.9% for m-5, 2.3% for m-7, and 2.0% for m-9. Furthermore, in the stress–strain curves of the 3D model GG, the slope for m-5 was lower than those for m-7 and m-9. The elongation of PA11 in high-temperature L-PBF-LB/P was at least 40% [10]. Therefore, the mechanical properties of the products manufactured using the proposed method require further improvement.

**Figure 17:**
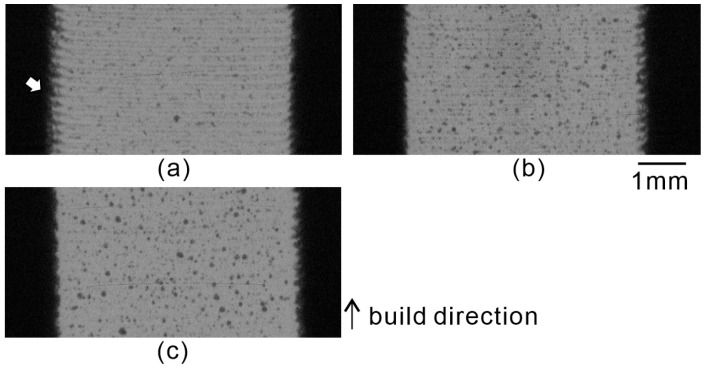
X-ray CT scanning images of specimens: (**a**) m-5, (**b**) m-7, (**c**) m-9. White arrows point to the regions where interlayer bonding was insufficient.

To clarify the factors behind the degradation of these mechanical properties, Figure 17 presents the X-ray CT scanning images of the tensile test specimens. In the figure, the gray areas represent PA11, whereas the black areas indicate air. Table 3 presents the evaluation results of voids obtained through image processing. The number of voids was lowest in m-5 and highest in m-7. The number of voids in m-9 was less than that in m-7. The average and maximum sizes of the voids were smallest in m-5 and largest in m-9. Therefore, clearly, among the three conditions, the porosity of the m-5 specimen was the lowest. It has been reported that the elongation of PA11 decreases with an increase in the porosity, which explains the greater elongation observed for m-5 [13]. From correlating the evaluation results of the voids with the decrease in elongation observed in the tensile tests with the increasing energy density of the irradiated laser, we found that the size of the voids, rather than their number, was a factor influencing the elongation. Furthermore, an examination of the left side of the m-5 specimen (white arrow in Figure 17) revealed that, although layers were present, there were regions where interlayer bonding was insufficient. Consequently, the actual bonded area was smaller than the apparent shape, which likely resulted in a lower slope for the m-5 specimen in the stress–strain curve of the 3D model GG specimen.

**Figure 18:**
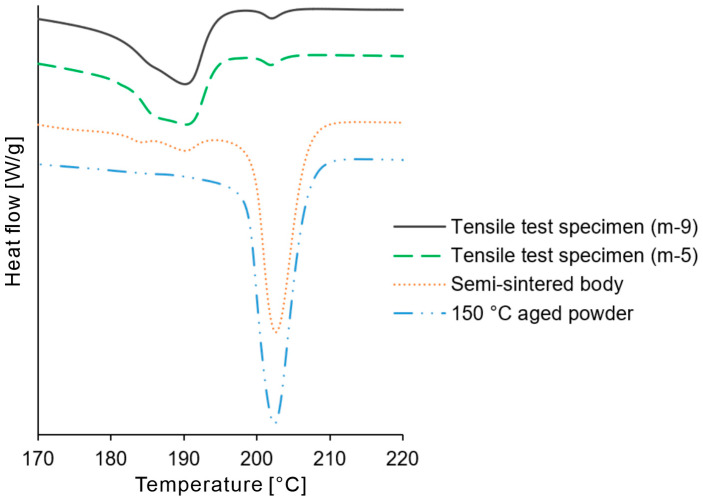
DSC curves of the tensile test specimen, semi-sintered body, and powder aged at 150 °C.

Figure 18 shows the DSC curves of the powder harvested from the 150 °C part cake, the semi-sintered body, and the 3D model GG specimen. The DSC curve of the powder harvested from the 150 °C part cake exhibited a single melting peak at approximately 202 °C. Similarly, the semi-sintered body also showed the largest peak at approximately 202 °C; however, in addition to this peak, it had smaller peaks at approximately 185 °C and 195 °C. These two smaller peaks appeared because of the partial melting of the powder particles under low-energy-density laser irradiation. The curve of the tensile test specimen built under the m-5 condition exhibited a peak at approximately 190 °C, with a clearly defined shoulder at approximately 185 °C. Similarly, the curve of the tensile test specimen built under the m-9 condition showed a peak at approximately 190 °C, with a shoulder at approximately 185 °C, although the shoulder was not as distinct as under the m-5 condition. Previous studies have reported that differences in the cooling rate of PA11 after melting can result in variations in the shoulder peak [27]. While the same report suggested that these differences could lead to variations in rigidity, the slope of the elastic region in the tensile tests using the 3D model EE was largely identical under the m-5, m-7, and m-9 conditions. Therefore, the differences in the shoulders of the peaks had a limited impact on the tensile test results. Both the m-5 and m-9 curves exhibited a minor peak at approximately 202 °C. This peak coincides with the melting point of the powder, suggesting the presence of residual unmelted powder. To eliminate this residual unmelted powder, a further increase in the laser energy density was required. However, the laser energy density of m-9 was near the upper limit and did not cause excessive surface melting. Moreover, from the perspective of reducing the elongation, an increase in the energy density was inappropriate.

It is necessary to improve these mechanical properties by enhancing interlayer bonding and eliminating the residual unmelted powder while suppressing thermal decomposition. Previous studies have demonstrated that strategic laser exposure can achieve mild heating, which may effectively suppress thermal decomposition [23]. Other studies have shown that it is possible to adjust the optical properties of the powders [31]. Adjusting the penetration depth may strengthen the bonding between the upper and lower layers while suppressing degradation. Combining these insights with semi-sintered low-temperature PBF-LB/P may allow for a precise control of the mechanical properties.

## 4. Conclusions and Future Work

This study demonstrated that low-temperature PBF-LB/P using semi-sintering can be executed without connecting the base plate and parts with support structures. The density of the built parts was nearly 1.00, achieving an apparent density comparable to that built by high-temperature PBF-LB/P. By applying warp corrections, we successfully fabricated tensile test specimens with a slice width of 68 mm. To the best of our knowledge, this slice width is currently the longest among low-temperature PBF-LB/P parts built without support structures. Furthermore, the warpage correction remained largely identical even under different laser conditions for melting. The tensile test results indicated that the elongation at break of the specimens decreased with an increase in the laser energy density. X-ray CT scanning and DSC analyses of the specimens revealed the presence of voids, insufficient interlayer bonding, and unmelted powder within the specimens. Overcoming these drawbacks is crucial for ensuring good mechanical properties, and it remains a challenge for future work.

In the future, it will be necessary to verify whether the proposed process can be established using various other powders. Moreover, the appropriate amount of semi-sintering required to achieve a successful build has not been verified. Future research should also clarify the recycling rate and potential for reusing semi-sintered powders.

## Figures and Tables

**Table 1:** Laser parameters for semi-sintering.

No.	s-1	s-2	s-3	s-4	s-5	s-6	s-7	s-8	s-9
Laser power [W]	4	5	6	7	8	9	10	11	12
Scan speed [mm/s]	10,000								
Scan pitch [mm]	0.09								
Energy density [10^−2^ J/mm^2^]	0.44	0.56	0.67	0.78	0.89	1.0	1.11	1.22	1.33

**Table 2:** Laser parameters for melting.

No.	m-1	m-2	m-3	m-4	m-5	m-6	m-7	m-8	m-9	m-10	m-11	m-12
Laser power [W]	4	5	6	7	8	9	10	11	12	13	14	15
Scan speed [mm/s]	4000											
Scan pitch [mm]	0.04											
Energy density [10^−2^ J/mm^2^]	2.50	3.13	3.75	4.38	5.00	5.63	6.25	6.88	7.50	8.13	8.75	9.38

**Table 3:** Evaluation results of the voids.

	m-5	m-7	m-9
Numbers of voids	55	201	142
Average size [10^−3^ mm^2^]	2.34	3.12	3.52
Maximum size [10^−3^ mm^2^]	14.6	14.8	17.4

## Data Availability

The raw data supporting the conclusions of this article will be made available by the authors on request.
